# Structural studies of elastic fibre and microfibrillar proteins

**DOI:** 10.1016/j.mbplus.2021.100078

**Published:** 2021-07-07

**Authors:** Mukti Singh, Mark Becker, Alan R.F. Godwin, Clair Baldock

**Affiliations:** Wellcome Centre for Cell-Matrix Research, Division of Cell-Matrix Biology and Regenerative Medicine, School of Biological Sciences, Faculty of Biology, Medicine and Health, University of Manchester, Manchester Academic Health Science Centre, Manchester M13 9PT, UK

**Keywords:** Fibrillin, Tropoelastin, SAXS, Electron microscopy

## Abstract

Elastic tissues owe their functional properties to the composition of their extracellular matrices, particularly the range of extracellular, multidomain extensible elastic fibre and microfibrillar proteins. These proteins include elastin, fibrillin, latent TGFβ binding proteins (LTBPs) and collagens, where their biophysical and biochemical properties not only give the matrix structural integrity, but also play a vital role in the mechanisms that underlie tissue homeostasis. Thus far structural information regarding the structure and hierarchical assembly of these molecules has been challenging and the resolution has been limited due to post-translational modification and their multidomain nature leading to flexibility, which together result in conformational and structural heterogeneity. In this review, we describe some of the matrix proteins found in elastic fibres and the new emerging techniques that can shed light on their structure and dynamic properties.

## Introduction

Elastic fibres endow connective tissues with their essential properties of elasticity and resilience and are essential for normal tissue function and homeostasis. Fibrillin microfibrils act as a template for elastin deposition during elastic fibre formation which is required to maintain the integrity of elastic tissues. These molecules have a complex hierarchical assembly, however new approaches are revealing important insights into their structural organisation and how their assembly supports their biological function.

## Elastic fibre proteins

### Elastin and fibrillin

Elastic fibres are essential components of all mammalian elastic tissues such as lung, skin, large diameter blood vessels and elastic cartilage. The main components of elastic fibres are elastin and fibrillin [1], however an array of matrix proteins are required for their correct assembly and function [2]. Elastic fibres are predominantly composed of elastin, which forms an insoluble core. Elastin is formed from the soluble precursor, tropoelastin, a 60 – 70 kDa monomeric protein secreted from various cell types such as fibroblasts and smooth muscle cells [3], [4]. Expression of tropoelastin is markedly high during mid-gestation in comparison to relatively low levels in adult tissues [5], [6]. Tropoelastin rapidly and spontaneously assembles from a monomer to n-mer in a process referred to as coacervation, and the assembled form is stabilised by cross-linking [7].

The domain arrangement of tropoelastin follows a distinct pattern of alternating hydrophobic and hydrophilic regions. The hydrophobic regions predominantly contain valine, proline and glycine residues, whereas the hydrophilic or cross-linking regions are characterised by repeating lysine-alanine and lysine-proline residues which can participate in the formation of desmosine cross-links essential for polymerisation of tropoelastin in elastic fibre assembly [8], [9]. Moreover, interactions between the hydrophobic regions of tropoelastin during coacervation results in the alignment of lysine residues which are in turn are enzymatically cross-linked by enzymes lysyl oxidase (LOX), LOX-like 1 (LOXL-1) and LOXL-2, aiding the formation of larger elastin aggregates crucial for elastic fibre formation [10]. This process is described in more detail in the following reviews [1], [7], [11].

**Fig. 1:**
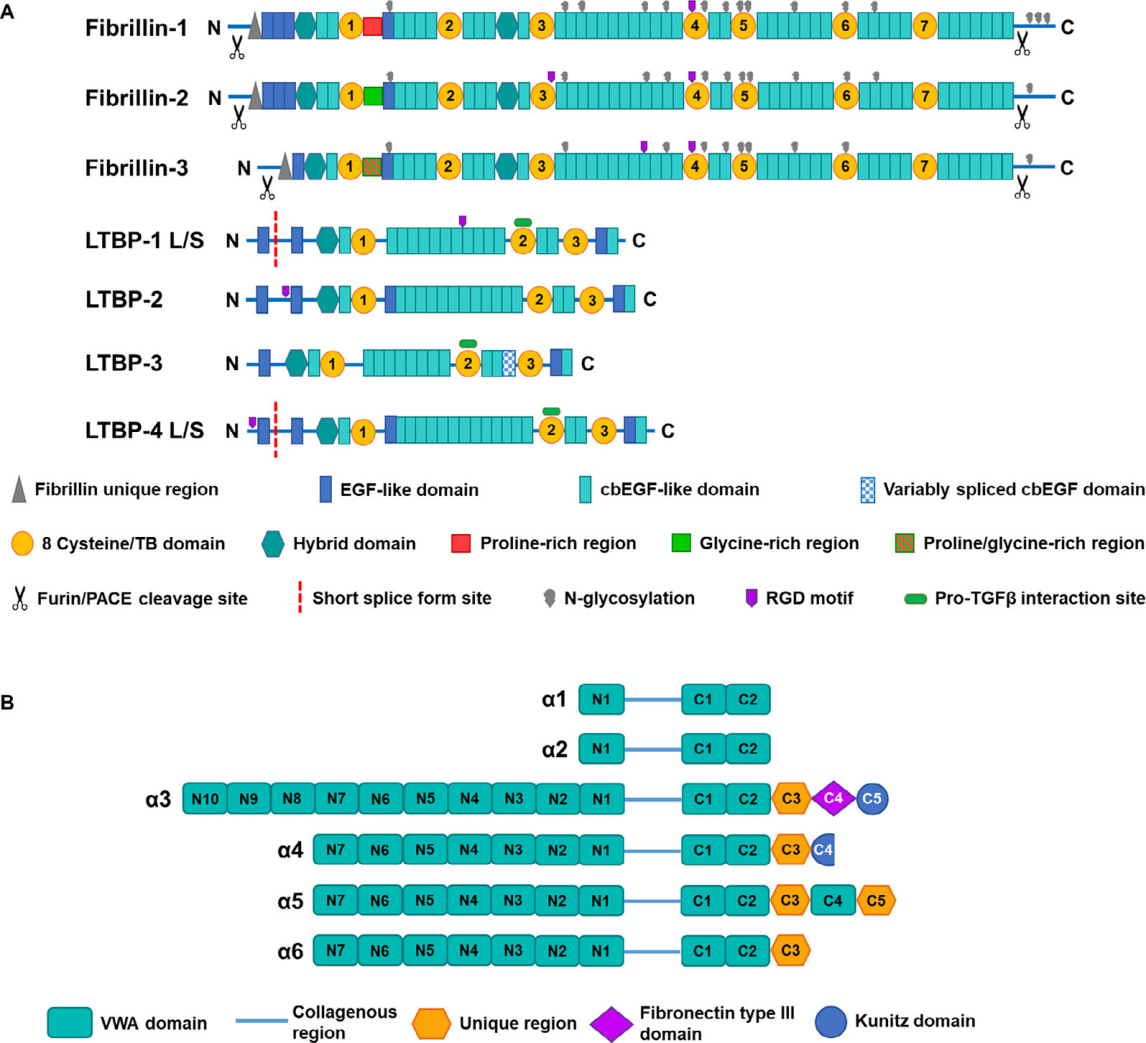
**Domain structures of the fibrillin/LTBP superfamily and collagen VI α-chains**. (A) Diagram showing the domain organisation of fibrillin-1-3, and LTBP-1-4. (B) Diagram showing the domain arrangement of α-chains 1-6 of collagen VI.

The other major component of elastic fibres is fibrillin which assembles to form microfibrils. There are three structurally homologous isotypes – fibrillin-1, −2 and −3 (Fig. 1). There are species and tissue dependant differences in the expression levels of the three isotypes, with fibrillin-1 being the predominant isotype found in adult human tissues [12], [13], [14], [15]. Secreted as pro-peptides, the N- and C-termini of all fibrillin isotypes are catalytically processed by furin to allow for microfibril formation [16], [17], [18], [19]. Fibrillin monomers are ~ 350 kDa multi-domain glycoproteins comprised of an array of interspersed epidermal growth factor-like (EGF) domains, TGFβ binding-like (TB) domains, and hybrid domains – all of which are essential for their functions. The EGF domains are the most abundant motif across all fibrillin isotypes. Despite all 3 fibrillin isotypes sharing structural homology, there are key differences between them. The N-terminal region of fibrillin-1 contains a proline-rich region, whereas fibrillin-2 contains a glycine-rich region and fibrillin-3 contains a proline-glycine rich region (Fig. 1). Investigations into the relevance of the proline-rich region in fibrillin-1 suggests that this domain confers hinge-like flexibility to fibrillin-1 [20]. All fibrillin isotypes are glycosylated, however they have different glycosylation states with fibrillin-1 having 15, fibrillin-2 having 12 and fibrillin-3 containing 10 predicted sites.

### LTBPs

There are many other components of elastic fibres, but the latent TGFβ binding proteins (LTBP)s are key components that support both elastic fibre assembly and cell signalling [21]. The LTBP family is comprised of four members (LTBP-1, −2, −3 and −4), each 150–220 kDa glycoproteins containing cbEGF repeats interspersed with TB domains (Fig. 1), and are members of the fibrillin/LTBP superfamily [22], [23], [24], [25]. Owing to alternate splicing of the N-terminus, LTBP-1 and −4 have long and short isoforms [25], [26], [27], where knockout mouse models of each variant results in tissue abnormalities or early fatalities [28], [29], [30]. LTBP-1 undergoes transglutaminase-2 (TG2) mediated multimerisation linking LTBP-1 monomers in N-terminus-to-N-terminus and N-terminus-to-C-terminus arrangements [31]. Despite belonging to the same family, LTBPs carry out different functions in the extracellular matrix (ECM). LTBP-1, −3 and −4 regulate the bioavailability of TGFβ [32], [33], [34], LTBP-2 is unable to covalently bind to latent TGFβ as it lacks the two amino acid insertion in the second TGFβ-binding like or TB domain [35], that is found in the other LTBPs. LTBP-2 and −4 also play a structural role in stabilising fibrillin-1 microfibrils in the ECM as well as elastogenesis but the specific structural or sequence differences that endow these functionalities are as of yet unknown [36], [37], [38], [39].

Other proteins involved in elastic fibre assembly include fibulins-4 and −5, A Disintegrin And Metalloprotease with Thrombospondin type-1 repeats (ADAMTS) and ADAMTS-Like (ADAMTSL) proteins and microfibril associated glycoproteins (MAGPs). MAGP-1 and −2 (also known as microfibrillar associated proteins (MFAPs)-2 and −5, respectively) copurify and colocalise with microfibrils [40], [41]. Neither are essential for elastic fibre assembly but promote elastin deposition onto microfibrils [42], [43]. MFAP-4 also enhances elastin assembly and binds to and colocalises with fibrillin-1 [44]. A subset of ADAMTS and ADAMTSL proteins have been implicated in microfibril assembly and their association with fibrillin-1 related pathologies suggests they modulate fibrillin-1 function [45]. ADAMTS10 cleaves fibrillin-2 and supports biogenesis of fibrillin-1 microfibrils [46], [47], [48], whereas ADAMTS6 inhibits microfibril deposition [49] while ADAMTS17 may support secretion and assembly of fibrillin-1 [50]. ADAMTSL2 interacts with LTBP-1 so may play a role in regulating TGFβ availability [51]. ADAMTSL4 supports fibrillin microfibril deposition in ciliary zonules [52]. Fibulin-4 and −5 are extracellular glycoproteins that have roles in elastogenesis [53], [54], [55]. They bind to both tropoelastin and fibrillin and support lysyl oxidase mediated elastin cross-linking [53], [55], [56], [57]. The role of LTBP-4 in elastogenesis is supported by direct interactions with fibulin-4 and −5 [37], [58].

### Collagen VI - a microfibril-forming protein

In addition to fibrillin microfibrils, collagen VI is associated with elastic fibres in many tissues and forms beaded microfibrils important in the maintenance of the structural integrity of connective tissues [59]. There are six collagen VI α chains, α1-6 [60], [61], [62]; chains α1, 2, 3 and 6 are widely expressed throughout most tissues [63] with the α5 chain having a more restricted expression pattern and is found in skin, lung, testis and colon [64], [65]. In humans and chimpanzees, the α4 chain is not functional and is not translated [61]. Collagen VI α-chains have relatively short collagenous regions surrounded by globular domains which consist of arrays of von Willebrand Factor type A (vWFA) domains which have been implicated in protein–protein interactions [66], [67], [68]. The C3 domain in the α3 chain shares homology with type III fibronectin domains and both α3 and α4 chains have C-terminal Kunitz-like domains [69], [70] (Fig. 1). The α1 and α2 chains are a similar size and domain structure and chains 3–6 have longer N-terminal vWFA arrays and are more similar in structure to one another [61], [62].

Three collagen VI α-chains form heterotrimeric “monomers” consisting of an α1 and α2 chain and the third chain can be any of the long alpha-chains (α3-α6). Monomers form disulphide linked anti-parallel dimers before associating to form tetramers which are secreted into the extracellular space. Collagen VI tetramers then form beaded microfibrils in an overlapping end-to-end assembly [71], [72]. Collagen VI microfibrils form higher order assemblies in a tissue specific manner. In skin, collagen VI microfibrils form web-like networks and associate with banded collagen II and III fibrils [63]. Collagen is also found in high concentration in the pericellular matrix where it forms a basket like meshwork surrounding chondrocytes in articular cartilage [73], [74], [75]. In the pericellular matrix, the globular regions of collagen VI microfibrils associate to form node structures ~ 30 nm in diameter [76]. The formation of these nodes is potentially supported through interaction of the small leucine rich proteoglycan biglycan which has been shown to form large hexagonal lattice like structures when incubated with collagen VI *in vitro* [77].

## Structural analysis of elastic fibre proteins

### High-resolution structural analysis by X-ray crystallography and NMR

**Fig. 2:**
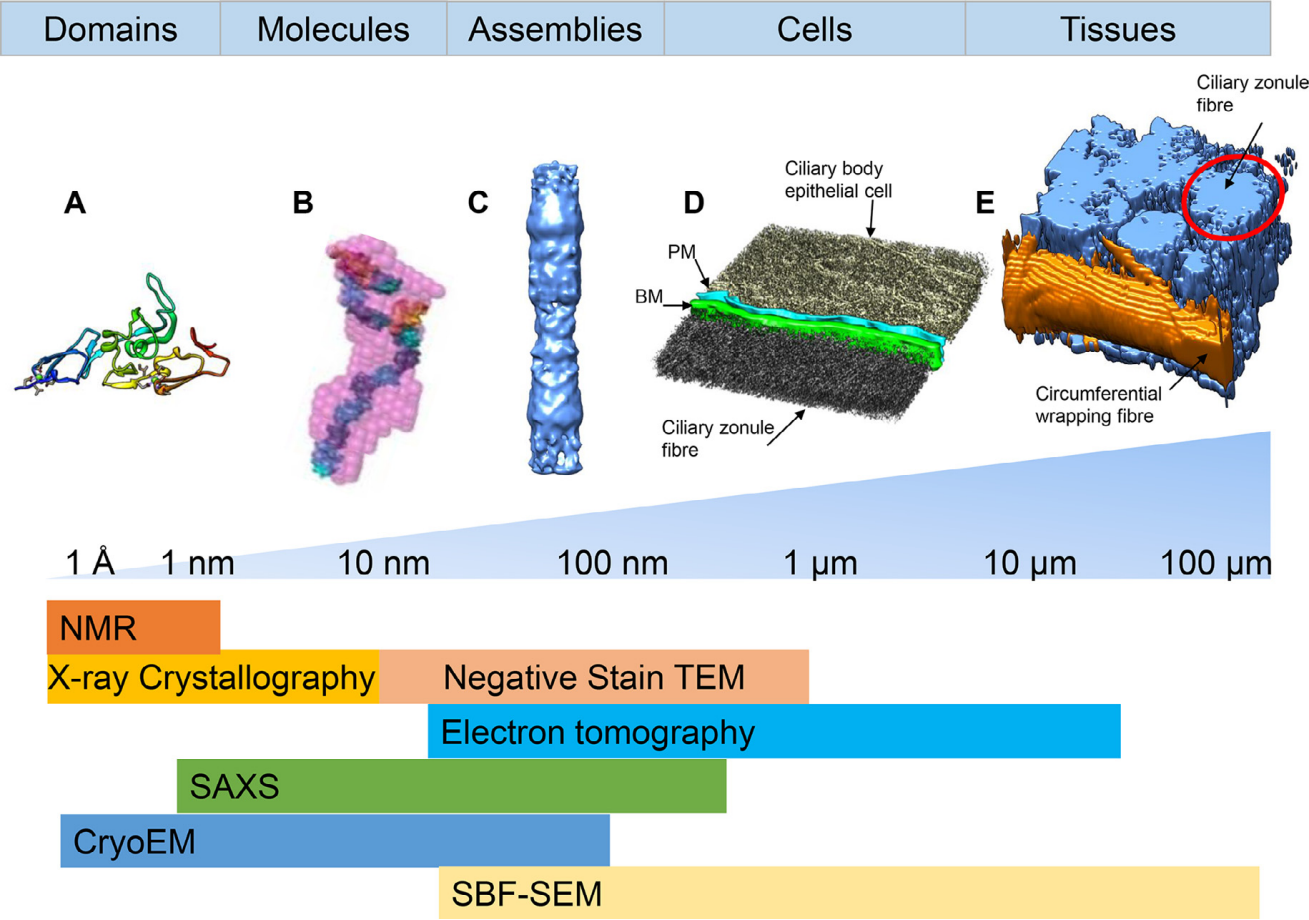
**Structural techniques used for studying the hierarchical organisation of fibrillin microfibrils**. Fibrillin-1 molecules have a complex hierarchical organisation from individual domains up to microfibrillar bundles in tissues. This figure highlights the range of techniques which have been used to study the organisation of fibrillin and the length scales which they can be used over. A) The high-resolution structure of fibrillin-1 domains cbEGF22-TB4-cbEGF23 determined by X-ray crystallography [82]. B) A bead model computed from SAXS data of a region of the fibrillin molecule (protein fragment 17 (PF17)(cbEGF16-22-TB4 cbEGF23-24-TB5-cbEGF25)) with homology models of domains docked into the density [91]. C) A negative stain structure of an extracted ciliary zonule fibrillin microfibril [109]. D) A 3D segmentation of an electron tomogram of a ciliary zonule fibre next to the ciliary body basement membrane (BM) which is coloured in green [109]. The ciliary body epithelial cell is shown in gold with its plasma membrane (PM) segmented in blue. E) Tissue organization of the ciliary zonule from an SBF-SEM dataset of a bundle of ciliary zonule fibres rendered in 3D. A ciliary zonule fibre (highlighted in orange) can be seen wrapping around a bundle of fibres (shown in blue). A single ciliary zonule fibre has been highlighted with a red circle [109].

Due to their size and modular, multidomain construction, which typically results in flexibility, extracellular matrix proteins do not lend themselves to conventional high-resolution structural biology techniques such as X-ray crystallography or NMR. Most matrix proteins have post-translational modifications, such as glycosylation which is another hurdle for these techniques. Therefore, thus far high-resolution structures determined via these conventional techniques are limited to single domains or short regions of the molecule (Table 1). Nevertheless, these structures have provided valuable insight into the folding of individual domains and has enabled the construction of models for arrays of domains. For fibrillin and LTBP1, a number of structures have been solved of their composite domains. EGF domains consist of a major and minor double stranded beta sheet which contains six cysteine residues which form three intra-domain disulphide bonds [78] (Fig. 2A). The majority of EGF domains in fibrillin are calcium-binding EGF domains and contain the consensus sequence (D/N)X(D/N)(E/Q)Xm(D/N)Xn(Y/F) [79]. NMR and X-ray crystallography studies have shown that cbEGF domains can form rod-like structures on binding of calcium through inter-domain interactions which contribute to structural stability [78], [80], [81]. TB domains (also known as 8-cysteine motifs), are unique to the fibrillin/LTBP superfamily and have a globular structure which consists of six β-strands and two α-helices which are stabilised by four disulphide bridges (Fig. 2A) [35], [82], [83]. The interaction between TB domains and their neighbouring EGF/cbEGF domains are thought to provide fibrillin molecules with a degree of flexibility. TB4 in fibrillin-1 contains a flexible loop with an RGD site which is involved in integrin binding [82]. All fibrillin isotypes contain two hybrid domains that possess N-terminal homology to TB domains and C-terminal homology to EGF/cbEGF domains [84]. The hybrid domains have one β-sheet formed from three β-strands in the N-terminal half of the domain and two β-strands in the C-terminal half and one α-helix. The first hybrid domain in fibrillin contains an unpaired cysteine residue which was thought to be involved in lateral assembly of fibrillin monomers however fibrillin monomers missing the first hybrid domain can still form microfibrils [85]. The N-terminal region of fibrillin-1 is also involved in the sequestration of TGFβ through a bipartite interaction with the C-terminus of LTBP-1 [86], [87], [88]. The N-terminal domains EGF2-EGF3-Hyb1-cbEGF1 were solved by NMR [88]. This fragment has a linear structure and modelling of the LTBP-1-fibrillin interaction suggests how LTBP-1 can wrap around fibrillin microfibrils whilst also interacting with other matrix components.

**Table 1:** The domains/regions of fibrillin-1, LTBP1 and collagen VI resolved using different structural biology approaches. N.S. – not stated.

Protein	Domains	Structural approach	Resolution	Reference
Fibrillin-1	Fibrillin unique N-terminal region – EGF3	NMR	N.S.	[149]
	EGF2 – cbEGF1	NMR	N.S.	[88]
	cbEGF9 – hyb2 - cbEGF10	X-ray crystallography	1.8 Å	[84]
	cbEGF12-cbEGF13	NMR	N.S.	[81]
	cbEGF16 – cbEGF24	SAXS	~20 Å	[91]
	cbEGF22 –TB4- cbEGF23	X-ray crystallography	1.35 Å	[82]
	TB6	NMR	N.S.	[83]
	cbEGF32-33	NMR	N.S.	[78]
	Overlapping constructs from TB1 to C-ter furin site	SAXS	~20 Å	[90]
LTBP1	TB2	NMR	N.S.	[35]
	Overlapping constructs for LTBP1S	Negative stain EM and SAXS	~20 Å	[31]
Collagen VI	α4; α5; α6 N-ter regions α1; α2; α4; α6 C-ter regions	Negative stain EM and SAXS	~20 Å	[105]
	α3 N9 – N1	Negative stain EM and SAXS	~20 Å	[104]
	α3 Kunitz domain	X-ray crystallography	1.6 Å	[69]
		NMR	N.S.	[70]
	α3 N5	X-ray crystallography	1.2 Å	[67]
	α3 N2	X-ray crystallography	2.2 Å	[68]

Furthermore, knowledge of domain structures is valuable to support in silico analyses, computational approaches have gained popularity in recent decades as their methodologies have been refined to provide accurate insights into molecular structure and movement. Steered molecular dynamics simulations have shown that calcium binding in the cbEGF domains of fibrillin-1 decreases under mechanical stress which has the potential to contribute to fibrillin flexibility at low strain [89]. These findings support a mechanosensitive role for fibrillin where matrix strain could modulate calcium-binding to provide a switch where localised changes in structure could influence processes such as extracellular TGFβ activation [89].

### Nanoscale structural analyses

For structural insight into longer arrays of domains or whole molecules, lower resolution techniques, such as small angle X-ray scattering (SAXS) have been employed (Fig. 2). SAXS is a solution-based technique that is not restricted by the requirement for a protein crystal. SAXS is amenable to analysing longer arrays of domains and as well as providing shape information, details of molecular flexibility and hydrodynamic properties can be extrapolated. X-ray scattering studies of larger fibrillin fragments showed that arrays of cbEGF domains are flexible and can form non-linear conformations (Fig. 2B) [90], [91]. Analysis of the region containing the second hybrid domain of fibrillin-1, has shown that mutations occurring in this domain adversely affect protein folding [92]. SAXS studies of LTBP-1 showed that it has a compact N-terminus with flexible extended array of cbEGF domains and flexible C-terminus [31]. Recently, using atomic force microscopy (AFM) it was shown that LTBP-4L is secreted in a compact conformation and interaction with multimers of fibulin-4 induce a conformational change in LTBP-4L causing it to adopt a more elongated form [93], which was also observed in interactions with fibulin-5 multimers however to a lesser extent. Moreover, the elongated conformation of LTBP-4 imaged with AFM, is consistent with SAXS data which shows that the N-terminal region adopts an elongated rigid conformation and the C-terminus a flexible elongated structure [94], similar to LTBP-1 [31].

The nanoscale structure of human full-length tropoelastin and over-lapping N- and C-terminal fragments were analysed using SAXS and Small Angle Neutron Scattering (SANS) [95] which showed that the N-terminal region is a coiled region with spring-like functionality, and the C-terminal region forms a protruding foot containing the GRKRK motif to facilitate cell attachment via integrin binding [96]. The N- and C-terminal regions are connected by a bridge region encompassing a predicted hinge motif which endows the molecule with a degree of flexibility [97], [98], [99]. The SAXS-based bead models of tropoelastin have formed the basis of elastic network models to elucidate the relationship between local and global structures and the dynamics of tropoelastin by defining the molecular motions intrinsic to the protein. Sub-molecular secondary structural changes can be predicted after perturbation of the hinge region which were cross-validated with wet-lab experimentation [100].

With advances in computational power and software, full-atomistic modelling of protein structure is achievable using approaches such as replica exchange molecular dynamics (REMD) simulations, an accelerated sampling method for molecular dynamics. The full-atomistic model of tropoelastin was developed based on REMD simulations [101]. This model revealed that despite its flexible nature tropoelastin maintains a canonical or “average” structure, based on the distribution of its possible conformations. Structures can be cross-validated against biophysical and hydrodynamic data, and the canonical tropoelastin structure was determined to be highly similar to the bead model calculated from SAXS/SANS data. Furthermore, the atomistic model uncovered the contributions of each molecular region to the flexibility of the tropoelastin. For example, the cell-interactive C-terminus was found to be highly flexible which could support the interactions between tropoelastin and integrin receptors. Computational models have also been used to predict the molecular consequences of synthetic and disease-causing mutations of tropoelastin. For example, models involving single point mutations at negatively charged residues demonstrated both regional and global destabilization of the structure of tropoelastin, which were validated by SAXS [99], [101].

These analyses can be extended to complexes, where the SAXS analysis of a fibrillin-tropoelastin complex, cross-linked by TG2, showed that the complex was formed from an end-to-end assembly but retained features of the individual proteins. Elastic network models were constructed using the SAXS bead models to compare the dynamics of tropoelastin and fibrillin individually as well as in the cross-linked complex. These data indicated that tropoelastin is less mobile when bound to fibrillin, and this molecular stabilisation extends along the length of the tropoelastin molecule to regions remote from the cross-linking site. Together, these data suggest a long-range stabilising effect of cross-linking that occurs due to the covalent linkage of fibrillin to tropoelastin which suggests that this interaction stabilises the elastin precursor so it is primed for elastic fibre assembly [102].

A combined approach of multiple low-resolution structural techniques provides further confidence in the models predicted, for instance a combination of SAXS and negative stain TEM with single particle analysis revealed structural features of LOXL2 which mediates cross-linking of tropoelastin [103]. Furthermore, SAXS and single particle analysis of the N-terminal vWFA domains of the collagen VI α3 chain reveal these domains to adopt a compact C-shaped globular structure [104]. The N-terminal domains of the α4, α5 and α6 chains also have a C-shaped structure [105]. Furthermore, SAXS measurements and *ab initio* modelling of the C-termini of α1, α2, α4 and α6 determined these domains to be more elongated and flexible [105].

### Structural analysis of microfibrillar assemblies

To image the assembled microfibrillar forms of elastic fibre proteins such as fibrillin microfibrils, electron microscopy has long been the technique of choice. A number of studies have provided details of fibrillin microfibrils showing their beads-on-a-string appearance with ~ 56 nm periodicity and diameter of 10–12 nm [106], [107], [108] (Fig. 2C). Comparing data from different approaches it can be surmised that they are hollow tube-like fibrils which when imaged in cross section have eight molecules in a ring-like structure. Microfibrils have a distinct asymmetric banding pattern and can be sub-divided into regions which have been named the bead, arm, interbead and shoulder regions [20]. Structural studies to determine the nanostructure of fibrillin microfibrils using negative-stain TEM and single particle analysis [109], revealed greater detail of how fibrillin is organised in mature microfibrils. The dense bead region has an outer ring with a complex interwoven core which is connected to four separate arm regions. The arm regions extend into compacted interbead region and a more flexible shoulder region. Epitope labelling of microfibrils suggest fibrillin-1 monomers align in a polar N-terminal to C-terminal fashion with the N- and C-termini on opposite sides of the bead [110]. Later it was reported that fibrillin molecules interact laterally [111], [112], [113], creating a lattice of eight molecules consistent with a mass of ~ 2500 kDa determined by scanning transmission electron microscopy mass mapping [114], which are further cross-linked via TG2 [115]. Fully extended fibrillin molecules are ~ 150 nm long [116] so to account for a 56 nm repeating microfibrillar structure two popular models for fibrillin arrangement have been proposed; a staggered alignment where fibrillin molecules span two [117] or three periods [82] or a molecule folding model where a fibrillin molecule folds back on itself and spans only a single repeat [90].

To analyse higher order assembly, techniques such as electron tomography or serial block-face scanning electron microscopy (SBF-SEM) imaging have been used. The 3D hierarchical organisation of fibrillin microfibrils in bovine ciliary zonule was illustrated using SBF-SEM and electron tomography [109]. Individual fibrillin microfibrils could be resolved in tomograms (Fig. 2D) and could be correlated with the large interwoven zonule fibres observed with SBF-SEM with diameter of ~ 0.5 to 3.5 μm (Fig. 2E). The microfibrils in the zonule fibres have a spacing of ~ 28 nm and are held together by protein bridges and further supported by smaller diameter bundles of microfibrils wrapping around their perimeter. This spacing has also been measured by X-ray scattering studies of ciliary zonule fibres which also showed microfibrils in ciliary zonules had a spacing of 28 nm [118]. These zonule fibres then form larger bundles which are held together by circumferentially wrapping zonule fibres in a fascicle-like organisation (Fig. 2E). The bridging proteins holding microfibrils together in zonule fibres have yet to be identified however LTBP-2 is a potential candidate for the bridging structures [119] and is the second most abundant protein in the ciliary zonule [120]. Furthermore, loss of LTBP-2 leads to disruption of the ciliary zonule [38]. SBF-SEM and X-ray scattering has also been used to resolve elastic fibres in corneal tissue to determine changes in the organisation of elastic fibres in Marfan Syndrome mouse models [121].

## A new era of structural biology for the analysis of extracellular matrix proteins

Cryogenic electron microscopy (cryoEM) has held potential for the imaging of extracellular matrix proteins, where with single particle analysis (computationally combining individual particles to reconstruct three-dimensional maps) 3D structures can be determined [122]. Recent advances have made single-particle cryoEM an easily accessible and widely applicable technique heralding a new era for structural biology termed the “resolution revolution” [123]. One of the major advancements behind the resolution revolution was the development of direct electron detection cameras [124]. The new sensors enabled higher resolution electron detection, whilst also allowing for high frame rates, moving data collection from individual images to movie stacks. Recording movie stacks instead of individual images allows for motion correction of particle movement, induced by the electron beam [125]. Motion correction increases the resolution of the collected data and partially mitigates the radiation damage, induced by the electron beam [126], [127], [128]. This allows optimised data collection, maximising contrast and signal-to-noise without excessive radiation. The development of direct electron detection cameras allowed cryoEM structures to approach crystallographic resolutions, for larger proteins [129].

One of the challenges of cryoEM is the reconstruction of three-dimensional maps from two-dimensional data. This process requires a large number of images and is computationally demanding [130], [131]. The resulting resolution depends on the conformational homogeneity of the sample and the ability to capture a variety of different angles. Individual particles are sorted into 2D classes where initially a maximum likelihood probabilistic approach was used [132]. However, a Bayesian approach proved to be more robust and better suited for isolating subsets of conformations within heterogeneous samples such as conformationally flexible extracellular matrix proteins [133]. Electron microscopes and associated sample preparation has become increasingly automated which along with improved software has led to the automation of large parts of the data collection and processing pipeline [134]. Real-time pre-processing of data collected allows the experimenter to optimise data collection parameters and therefore enable the acquisition of high-quality data on a routine basis [135].

In recent years, cryoEM has helped solve structures of numerous new proteins and complexes, which were previously inaccessible by traditional structural biology techniques like X-ray crystallography and NMR. Instead of crystallising individual subunits and meticulously assembling their structures, the electron density maps of whole complexes can be solved [136], [137]. This also allowed the analysis of individual functional states of complex cellular machinery, like the spliceosome [137], [138]. Unsurprisingly, cryoEM has been widely utilised to study proteins and protein complexes present in the extracellular matrix. Using cryoEM, structural changes of collagen fibrils at mineralised hard/soft-tissue interfaces were studied, unveiling new mechanisms of tissue biomineralization [139]. Using a variation of cryoEM, cryo-scanning transmission electron tomography (CSTET), a three-dimensional map of native ECM was generated, giving a detailed insight into how the components of the matrix are organised, as well as giving insights into the macromolecular organisation of collagen VI microfibrils in tissues [140]. These data also revealed how cells respond to their microenvironment and how the matrix affects intracellular structures. CryoEM analysis has described the molecular structure of the bead region of collagen VI microfibrils. The microfibrils have a hollow head composed of four lobes connected to the collagenous interbead region and two C-shaped flexible tails. The bead region is thought to be composed of C-terminal vWFA domains from the three α-chains, whereas the tail region contains N-terminal domains [76]. Collagen VI can also form large banded aggregate structures which have been identified in the Bruch’s membrane of the eye from patients suffering from adult macular degeneration and Sorsby’s Fundus Dystrophy [141], [142], [143]. Large aggregates of collagen VI microfibrils have also been identified in the trabecular meshwork [144].

## Outlook

Recent and emerging advances enable the routine use of single-particle cryoEM in an increasing number of fields of structural biology. Being able to routinely generate high-resolution structures make cryoEM a suitable method for structure-based drug discovery, pushing further advances in pharmaceutical and medical research [145]. Technologies increasing resolution and contrast of samples make cryoEM suitable for studying smaller targets, such as proteases and regulators, controlling ECM formation and reorganisation, whilst novel methods to conduct time-based experiments allow the study of dynamic complexes. Improved image classification techniques are increasingly capable of sub-classifying individual conformational states in heterogeneous samples, which makes it possible to study complexes and processes with multiple different functional states [146], whilst other algorithms are able to reliably detect non-globular particles which is vital for the study of fibrillar ECM components [147], [148]. As a result, cryoEM still holds large potential for further advances in structure-based research, beyond traditional structural biology and makes cryoEM one of the driving technologies to improve our understanding of the structure and function of ECM proteins, complexes and processes.

## Declaration of Competing Interest

The authors declare that they have no known competing financial interests or personal relationships that could have appeared to influence the work reported in this paper.
